# Fatal renal diseases among patients with hematological malignancies: A population‐based study

**DOI:** 10.1002/jha2.99

**Published:** 2020-09-24

**Authors:** Sen Li, Kaixu Yu, Ying Chen, Wenjing Luo, Yongqiang Zheng, Yun Yang, Xue Yang, Xi Wang, Xiaolan Gao, Xindi Wang, Bian Wu

**Affiliations:** ^1^ Department of Urology, Union Hospital, Tongji Medical College Huazhong University of Science and Technology Wuhan China; ^2^ Department of Orthopedics, Tongji Hospital, Tongji Medical College Huazhong University of Science and Technology Wuhan China; ^3^ Department of Obstetrics and Gynecology, Tongji Hospital, Tongji Medical College Huazhong University of Science and Technology Wuhan China; ^4^ Department of Hematology, Union Hospital, Tongji Medical College Huazhong University of Science and Technology Wuhan China; ^5^ Cancer Center, Union Hospital, Tongji Medical College Huazhong University of Science and Technology Wuhan China; ^6^ Department of Ultrasound, Union Hospital, Tongji Medical College Huazhong University of Science and Technology Wuhan China; ^7^ Department of Breast and Thyroid Surgery, Tongji Hospital, Tongji Medical College Huazhong University of Science and Technology Wuhan China

**Keywords:** hematological malignancies, mortality, renal diseases, standardized mortality ratio

## Abstract

Patients with hematological malignancies might be at high risk for renal diseases as evidenced by earlier studies. We aim to investigate the mortality and risk factors of deaths due to renal diseases in this population. A total of 831 535 patients diagnosed with hematological malignancies in the Surveillance, Epidemiology, and End Results (SEER) database in the United States from 1975 to 2016 were identified. Standardized mortality ratio (SMR) was evaluated based on the general population's mortality data gathered by the National Center for Health Statistics. The mortality rate associated with renal diseases was 94.22/100 000 person‐years among patients with hematological malignancies (SMR = 3.59; 95% CI, 3.48‐3.70]). The highest mortality rate of dying from renal diseases was observed among multiple myeloma (MM) patients (307.99/100 000 person‐years; SMR = 7.98; 95% CI, 7.49‐8.50), followed by those with chronic myeloid leukemia (142.57/100 000 person‐years; SMR = 6.54; 95% CI, 5.63‐7.60) and chronic lymphocytic leukemia (103.66/100 000 person‐years; SMR = 2.51; 95% CI, 2.27‐2.77). The SMRs increased with time and were found to be the highest 10 years after cancer diagnosis. Independent predictors associated with death from renal diseases were found to be older age, male gender, blacks, unmarried, and MM, using the Cox proportional hazards model. We call for enhanced coordinated multidisciplinary care between hematologists and nephrologists to reduce the mortality rate of renal diseases among patients with hematological malignancies.

## 1 INTRODUCTION

Although the survival rate of patients with hematological malignancies has significantly improved in recent years, the incidence of kidney diseases among these patients is raising [1, 2]. These kidney diseases, which mainly include acute kidney injury (AKI), chronic kidney disease, proteinuria, nephrotic syndrome, and multiple myeloma (MM) nephropathy, are caused by the malignancy itself or by its treatment [3]. A set of Danish cohort studies including 37 267 people indicated that patients with MM, leukemia, and lymphoma were at 53%, 40%, and 31% risk of developing AKI within 5 years, respectively [4]. Another investigation comprising 163 071 cancer patients in Ontario, Canada evidenced that MM (26%) and leukemia (15%) patients exhibited a relatively high 5‐year incidence of developing AKI [5]. The occurrence of kidney diseases influences the treatment strategy, extends the period of hospital stay, decreases the patient's complete remission rate, and amplifies both treatment cost as well as mortality rate [6, 7, 8].

Earlier studies have investigated and categorized the pathogenesis of nephropathy among patients with hematological malignancies into three groups: the first category comprises nonspecific factors such as hypoperfusion; the second one involves the infiltration of tumors such as lymphoma or acute leukemia; and the third is related to therapies such as nephrotoxicity of chemotherapy and antibiotics [9]. In addition, cast nephropathy in patients suffering from MM may result in proteinuria and nephrotic syndrome owing to the deposition of light chain proteins [10].

This study was conducted to comprehensively analyze death due to renal diseases among patients with hematological malignancies involving a large population‐based cohort. The mortality rate due to renal diseases among patients with hematological malignancies was evaluated, and subgroups of patients linked to a greater risk of dying from renal diseases were identified.

## 2 PATIENTS AND METHODS

### 2.1 Data sources

This retrospective study was performed to identify patients suffering from hematological malignancies, the data for which were used from the Surveillance, Epidemiology, and End Results (SEER) database that documents information on cancer survival and incidence from population‐based cancer registries, covering approximately 26% of the US population [11]. The “public use” version of the database was opted, which comprised 18 registries starting from 1975 to 2016. To compare, the mortality data of the general US population from the National Center for Health Statistics spanning from 1969 to 2016 were used. This investigation was approved by the Institutional Review Board of the Tongji Medical College, Huazhong University of Science and Technology.

### 2.2 Patient population

A total of 831 535 patients diagnosed with hematological malignancies were included in this study. The exclusion criteria included: (a) information was exclusively collected from the death certificate, (b) autopsy only, and (c) lack of survival time. To exclude the influence of a secondary tumor on renal diseases, patients suffering from multiple primary tumors were eliminated in the type‐specific analysis. Only data for patients whose survival time was more than 100 000 person‐years have been presented. Therefore, data of patients suffering from acute monocytic leukemia, other acute leukemia, aleukemia, subleukemia, and not otherwise specific, other myeloid/monocytic leukemia, or other lymphocytic leukemia have not been presented.

### 2.3 Study variables

Variables extracted from the SEER database included sex (female and male); race (White, Black, and other including unknown); marital status (married; unknown; and unmarried including single, separated, divorced, widowed, unmarried, or domestic partner); age at diagnosis (0‐39, 40‐49, 50‐59, 60‐69, 70‐79, and 80+); type of hematological malignancies (MM and non‐MM); and the vital status at the last follow‐up (alive or dead). The survival time in certain patients, recorded as 0 months in the SEER database, was converted to one‐half of a month, although the patients did not survive for a full month. Moreover, the stage of cancer was not included in the study as the only stages of hematological malignancies are “distant” and “unstaged/unknown.”

Besides, patients coded as “nephritis, nephrotic syndrome, and nephrosis (50160)” according to the International Statistical Classification of Diseases and Related Health Problems; the 10th revision (ICD‐10) that included glomerular diseases (N00–N07), renal failure (N17–N19), and other disorders of the kidney and ureter (N25–N27); the 9th revision (ICD‐9, 580–589); and the 8th revision (ICD‐8, 580–584) were considered to have died due to renal diseases [ 1].

**FIGURE 1:**
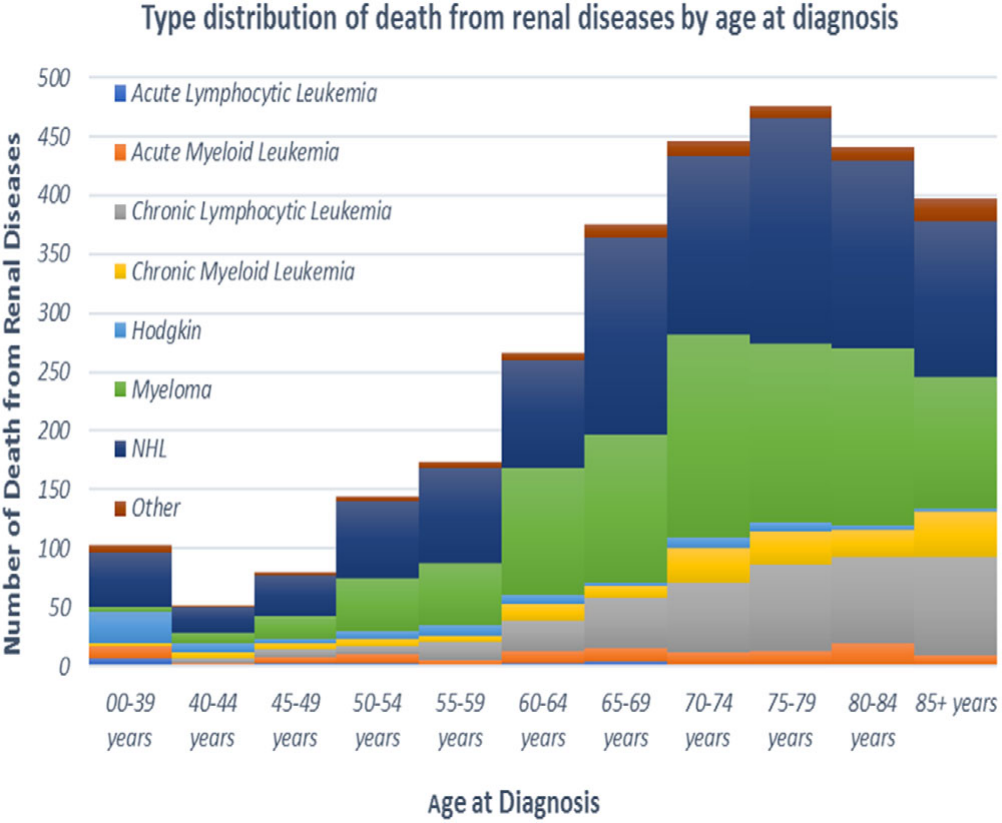
Site distribution of deaths due to renal diseases among patients with hematological malignancies in SEER 18 registries as a function of age at diagnosis

### 2.4 Statistical analysis

The number of deaths caused by renal diseases divided by person‐years of survival was calculated as the mortality rate among cancer patients listed in the SEER database. These data of cancer patients were compared with that of the general population with similar characteristics such as age, sex, and race distribution. The standardized mortality ratios (SMRs) and 95% confidence intervals (95% CIs) were computed as described earlier [11]. Five‐year age ranges were utilized for standardization. SMRs are not comparable with each other, as the standard population may differ among subgroups [12]. To detect the risk factors linked to mortality caused by renal diseases among cancer patients, we developed a multivariate Cox proportional hazards model. Observations were censored if the patient did not die of renal diseases at the time of the last follow‐up [ 1‐4].

**TABLE 1:** Mortality due to renal diseases in patients with hematological malignancies by demographic characteristics

Characteristic	Number of patients with cancer (%)	Number of observed deaths from renal diseases (%)	Person‐years	Mortality rate^a^	SMR^b^ (95% CI)
Age					
0‐39	114 793 (14%)	114 (3%)	1 062 514.54	10.73	19.76 (16.44‐23.74)
40‐49	70 934 (9%)	156 (4%)	514 294.50	30.33	11.25 (9.62‐13.16)
50‐59	125 305 (15%)	392 (10%)	772 216.83	50.76	7.24 (6.56‐8.00)
60‐69	178 854 (22%)	844 (21%)	880 814.75	95.82	5.16 (4.83‐5.52)
70‐79	195 206 (23%)	1300 (32%)	715 015.58	181.81	3.56 (3.37‐3.76)
80+	146 443 (18%)	1199 (30%)	308 646.71	388.47	2.34 (2.21‐2.47)
Sex					
Female	370 089 (45%)	1649 (41%)	1 960 089.08	84.13	3.58 (3.41‐3.76)
Male	461 446 (55%)	2356 (59%)	2 293 413.83	102.73	3.60 (3.46‐3.75)
Race					
Black	77 973 (9%)	709 (18%)	351 058.04	201.96	4.82 (4.47‐5.18)
Other	57 061 (7%)	208 (5%)	274 809.33	75.69	4.40 (3.84‐5.04)
White	696 501 (84%)	3088 (77%)	3 627 635.54	85.12	3.35 (3.24‐3.47)
Year					
1975‐1985	68 402 (8%)	361 (9%)	515 924.67	101.76	4.60 (4.15‐5.10)
1986‐1995	101 447 (12%)	509 (13%)	748 663.58	84.38	3.63 (3.33‐3.96)
1996‐2006	279 214 (34%)	1791 (45%)	1 833 379.79	97.69	3.79 (3.62‐3.97)
2007‐2016	382 472 (46%)	1344 (34%)	1 155 534.88	87.46	3.17 (3.00‐3.34)
Marital status					
Married	435 993 (52%)	2017(50%)	2 305 815.63	87.47	3.37 (3.23‐3.53)
Unknown	58 168 (7%)	297 (7%)	292 349.42	101.59	3.05 (2.72‐3.41)
Unmarried	337 374 (41%)	1691 (42%)	1 655 337.88	102.15	4.02 (3.84‐4.22)
All	831 535	4005	4 253 502.92	94.16	3.59 (3.48‐3.70)

Abbreviations: CI, confidence interval; SMR, standardized mortality ratios.

^a^ Per 100 000 person‐years.

^b^ The SMRs were calculated as the ratios of observed to expected number of deaths. The observed values represented the number of deaths due to renal diseases in patients with hematological malignancies, and the expected values represented the number of individuals who died of renal diseases in general population, with the same distribution of age, sex, and race.

**TABLE 2:** Mortality due to renal diseases in patients with hematological malignancies by type and years since diagnosis

	Time since cancer diagnosis			
Type^a^	0‐1 Year	1‐5 Years	5‐10 Years	>10 Years
Myeloma				
Number of deaths	388	373	139	49
Person‐years	74 059	157 222	60 324	21 811
SMR	10.26	5.84	8.52	14.57
95% CI	9.29‐11.33	5.27‐6.46	7.21‐10.06	11.01‐19.28
Acute lymphocytic leukemia				
Number of deaths	6	10	0	4
Person‐years	25 741	70 077	56 097	77 778
SMR	6.31	9.74	0	14.18
95% CI	2.83‐14.04	5.24‐18.10	0	5.32‐37.77
Acute myeloid leukemia				
Number of deaths	61	24	5	12
Person‐years	28 930	44 603	26 891	24 466
SMR	7.77	4.88	3.46	16.28
95% CI	6.05‐9.99	3.27‐7.29	1.44‐8.31	9.25‐28.67
Chronic myeloid leukemia				
Number of deaths	63	62	25	20
Person‐years	22 168	52 944	29 023	16 719
SMR	7.53	4.63	6.57	20.98
95% CI	5.88‐9.64	3.61‐5.94	4.44‐9.72	13.53‐32.52
Hodgkin lymphomas				
Number of deaths	19	20	15	34
Person‐years	43 630	138 230	119 118	160 393
SMR	4.92	2.45	3.44	13.16
95% CI	3.17‐7.71	1.58‐3.80	2.07‐5.71	9.40‐18.41
Non‐Hodgkin lymphomas				
Number of deaths	348	313	254	229
Person‐years	218 522	568 501	383 906	291 968
SMR	4.17	1.81	3.15	7.66
95% CI	3.76‐4.64	1.62‐2.02	2.79‐3.56	6.73‐8.72
Chronic lymphocytic leukemia				
Number of deaths	71	160	100	59
Person‐years	57 713	165 393	101 077	56 548
SMR	2.10	2.01	2.97	5.53
95% CI	1.66‐2.65	1.72‐2.34	2.44‐3.62	4.28‐7.14
Others^b^				
Number of deaths	44	23	10	13
Person‐years	14 658	32 057	21 285	20 525
SMR	7.15	2.90	3.00	8.70
95% CI	5.32‐9.61	1.93‐4.37	1.61‐5.58	5.05‐14.99
All				
Number of deaths	1000	985	548	420
Person‐years	485 422	1229.027	797 720.2	670 206
SMR	5.49	2.79	3.80	8.39
95% CI	5.16‐5.84	2.62‐2.97	3.50‐4.14	7.62‐9.23

Abbreviations: CI, confidence interval; SMR, standardized mortality ratios.

^a^ This analysis was limited to the patients only with hematological malignancies, and types of hematological malignancies with the follow‐up time greater than 100 000 person years were displayed.

^b^ Others comprised acute monocytic leukemia, other acute leukemia, aleukemia, subleukemia, and not otherwise specific, other myeloid/monocytic leukemia, and other lymphocytic leukemia.

**TABLE 3:** Mortality due to renal diseases in patients with hematological malignancies by type

Type^a^	Number of deaths	Number of patients	Person‐years^b^	Mortality rate^c^	SMR (95% CI)
Myeloma	949	94 524	308 123.8	307.99	7.98 (7.49‐8.50)
Chronic myeloid leukemia	170	27 289	119 236.5	142.57	6.54 (5.63‐7.60)
Chronic lymphocytic leukemia	390	64 765	376 244.6	103.66	2.51 (2.27‐2.77)
Acute myeloid leukemia	102	55 513	123 221.0	82.78	6.96 (5.73‐8.45)
Non‐Hodgkin lymphomas	1144	276 375	1 447 039.5	79.06	3.17 (2.99‐3.36)
Hodgkin lymphomas	88	48 207	457 964.6	19.22	4.70 (3.81‐5.79)
Acute lymphocytic leukemia	20	30 456	227 774.5	8.78	7.62 (4.92‐11.81)
Others^d^	90	24 360	87 544.6	102.80	4.55 (3.70‐5.60)

Abbreviations: CI, confidence interval; SMR, standardized mortality ratios.

^a^ The patients only with single primary tumors were included in the type‐specific analysis.

^b^ The data of patients with at least 100 000 person years were presented.

^c^ Per 100 000 person‐years.

^d^ Others comprised acute monocytic leukemia, other acute leukemia, aleukemia, subleukemia, and not otherwise specific, other myeloid/monocytic leukemia, and other lymphocytic leukemia

**TABLE 4:** Multivariable Cox regression analyses of deaths due to renal diseases in patients with hematological malignancies

	All deaths from renal diseases		
Variable	HR	95% CI	*P*‐value
Age at diagnosis, years	1.072	1.069‐1.074	<.001
Sex			
Male			Ref.
Female	0.634	0.593‐0.677	<.001
Race			
White			Ref.
Black	2.363	2.169‐2.574	<.001
Other	1.04	0.903‐1.197	.585
Marital status			
Married			Ref.
Unknown	1.049	0.928‐1.186	.445
Unmarried	1.399	1.306‐1.499	<.001
Year			
1975‐1985			Ref.
1986‐1995	0.929	0.808‐1.068	.298
1996‐2006	1.223	1.081‐1.382	.001
2007‐2016	1.081	0.951‐1.228	.235
Type			
Nonmultiple myeloma			Ref.
Multiple myeloma	2.653	2.472‐2.848	<.001

Abbreviations: CI, confidence interval; HR, hazard ratio.

All statistical tests were two sided, and the values with *P* < .05 were considered to be statistically significant. The SEER*Stat version 8.3.6 and the R version 3.51 statistical software packages were used for all analyses.

## 3 RESULTS

Thus analyzed data revealed that 4005 deaths occurred due to renal diseases among 831 535 patients suffering from hematological malignancies followed for 4 253 502 person‐years (Table 1). The mortality rate was 94.16/100 000 person‐years, and the equivalent mortality rate among the general US population was 26.23/100 000 person‐years, which resulted in an SMR of 3.59 (95% CI, 3.48‐3.70). The mean follow‐up time was 5.12 years (range = 0.50‐41.92 years).

### 3.1 Characteristics linked to higher mortality rate due to renal diseases

As patients suffering hematological malignancies grew older, the mortality rate due to renal diseases raised, but the SMR was observed to decrease gradually. The highest mortality rate was found to be recorded among patients above 80 years of age (388.47/100 000 person‐years), and the SMR to be the highest among patients up to the age of 39 years (SMR = 19.76; 95% CI, 16.44‐23.74). Among the survivors with hematological malignancies, a higher mortality rate due to renal diseases was recorded in men (SMR = 3.60; 95% CI, 3.46‐3.75), Blacks (SMR = 4.82; 95% CI, 4.47‐5.18), unmarried patients (SMR = 4.02; 95% CI, 3.84‐4.22), and patients diagnosed from 1975 to 1985 (SMR = 4.60; 95% CI, 4.15‐5.10).

### 3.2 Mortality risk due to renal diseases overtime postdiagnosis

The SMRs reportedly increased with time from diagnosis among the survivors suffering from hematological malignancies overall (Table 2), and were found to be the highest 10 years after the initial diagnosis (SMR = 8.39; 95% CI, 7.62‐9.23).

### 3.3 Type of hematological malignancies linked to higher mortality rate due to renal diseases

Majority of deaths due to renal diseases resulted among patients with non‐Hodgkin's lymphomas, MM, and chronic lymphocytic leukemia (CLL) accounting for 84% of the total deaths (Figure 1). MM patients exhibited the highest mortality rate due to renal diseases (307.99/100 000 person‐years; SMR = 7.98; 95% CI, 7.49‐8.50), followed by those that occurred due to chronic myeloid leukemia (CML) (142.57/100 000 person‐years; SMR = 6.54; 95% CI, 5.63‐7.60) and CLL (103.66/100 000 person‐years; SMR = 2.51; 95% CI, 2.27‐2.77; Table 3).

### 3.4 Factors linked to death due to renal diseases

Multivariate Cox regression analysis proved that MM (hazard ratio [HR] = 2.653; 95% CI, 2.472‐2.848; *P* < .001), increasing age (HR = 1.072; 95% CI, 1.069‐1.074; *P* < .001), and diagnosis between 1996 and 2006 (HR = 1.223; 95% CI, 1.081‐1.382; *P* = .001) were independent predictors of death from renal diseases among patients suffering from hematological malignancies (Table 4). In addition, Black race (HR = 2.363; 95% CI, 2.169‐2.574; *P* < .001) and unmarried status (HR = 1.399; 95% CI, 1.306‐1.499; *P* < .001) were also factors found to be strongly linked to deaths due to renal diseases among these patients. Meanwhile, female gender (HR = 0.634; 95% CI, 0.593‐0.677; *P* < .001) can be considered to be a protective factor compared with male gender for deaths caused by renal diseases.

## 4 DISCUSSION

Earlier studies have shown a higher incidence of renal diseases among patients suffering from hematological malignancies; however, the risk of deaths due to renal diseases among this population has not been explored much. This study first provides evidence that the risk of death due to renal diseases among patients with hematological malignancies is about 3.6 times that of the general US population, and recognized the groups of patients linked to higher mortality risk owing to renal disease.

Several studies have investigated the pathogenesis of renal diseases among patients suffering from hematological malignancies. Canet et al evidenced that renal diseases among patients suffering from hematological malignancies were caused due to direct drug‐induced nephrotoxicity as well as the complications of the disease [9]. Hypoperfusion and infection are common complications among patients suffering from hematological malignancies, and could lead to acute tubular necrosis, one of the major reasons for renal diseases among patients [7, 13]. In addition, for patients suffering renal diseases postcancer diagnosis, nephrotoxic antibiotics against infection and nephrotoxic chemotherapy drugs against hematological malignancies are known to worsen the renal diseases [14].

Patients with all types of hematological malignancies, especially MM, were found to be at a higher risk of death from renal diseases than the general US population. The incidence of MM has not been recorded to increase significantly of late, although the incidence of renal failure among patients suffering MM has tripled [1, 15, 16]. The multivariate analysis also indicated that MM patients were at a higher risk of death due to renal diseases than patients with other types of hematological malignancies. Monoclonal light chain proteins were the major cause of nephropathy among MM patients, which had toxic effects on glomeruli and tubules, and renal damage in MM most often was found to be tubular nephropathy [9, 17‐19]. Dehydration, hypercalcemia, sepsis, and nephrotoxic drugs have also been demonstrated to lead to the progression of renal diseases among MM patients [20, 21, 22]. In addition, CML patients exhibited a significantly high mortality rate due to renal diseases. This is attributable to the development of supportive care and chemotherapy such as tyrosine kinase inhibitors, which aid in prolonging the survival time of such patients [23, 24]; therefore, patients were more likely to die due to renal diseases.

Once diagnosed, the risk of death due to renal diseases was found to increase overall among patients suffering from hematological malignancies. This was perhaps due to the longer survival time that led to greater use of the nephrotoxic drugs by patients, which in turn subjected them to a higher risk of death due to renal diseases [1]. Furthermore, a higher age has also been shown to quicken the progression of renal diseases among these cancer survivors [25]. Noteworthy is the first year of cancer diagnosis that represented a greater risk of death due to renal diseases for the majority of hematological malignancies except all the rest. This perhaps is caused by the aggressive treatment imparted immediately following the cancer diagnosis.

The risk of patients suffering from hematological malignancies dying due to renal diseases raised with age at diagnosis, although SMR was found to decrease steadily. This may be due to the higher risk of death from cancer than that from renal diseases for elderly patients with hematological malignancies; therefore, most elderly patients had died due to hematological malignancies prior to dying from renal diseases. This multivariate analysis evidences that Blacks are at a higher risk of death due to renal diseases, which may be linked to the difference between the treatment options opted in distinct races. The differences in the risk of death due to renal diseases between different races are attributable to the economic status, medical level, and genetic factors, whereas the specific mechanism is yet to be studied further.

Our study has several limitations, most of which are related to the SEER database. First, the database is deficient of accurate data regarding chemoradiotherapy, which renders it difficult to assess the influence of chemoradiotherapy on the prognosis of cancer patients. Second, the reasons for death documented as “Nephritis, Nephrotic Syndrome, and Nephrosis” comprised glomerular diseases, renal failure, other kidney disorders, and ureter. Accurate evaluation of whether the patient died from renal diseases or ureteral diseases could not be made. Third, due to a short follow‐up period, the risk of death due to renal diseases among patients diagnosed with hematological malignancies in recent decades was seriously miscalculated. In spite of these limitations, this study is the first large sample‐ and population‐based study on the risk of death due to renal diseases in patients suffering hematological malignancies. Hence, the results of this study are considered to be reliable and applicable to the rest of the population.

In conclusion, our results indicated that all patients with hematological malignancies are at an increased risk of death due to renal diseases, which was found to rise from the time of cancer diagnosis. Hematologists should detect cancer patients with the abovementioned high‐risk factors at the earliest and focus more on the renal functions of patients during the cancer diagnosis, treatment for cancer, and follow‐up period posttreatment. Enhanced coordinated multidisciplinary care between hematologists and nephrologists becomes indispensable in case of the patients who have already developed renal diseases to reduce the mortality rate of renal diseases.

## AUTHOR CONTRIBUTIONS

SL designed research. KY, CY, WJ, and YZ calculated data. YY, XY, XW, XG, and XW analyzed the result. SL and KY wrote the paper. All authors revised the final version.

## CONFLICT OF INTEREST

The authors declare no conflict of interest.

## Data Availability

The data that support the findings of this study are available in SEER at: http://www.seer.cancer.gov, reference number [11]. These data were derived from the following resources available in the public domain: http://www.seer.cancer.gov.
